# ILDR2 has a negligible role in hepatic steatosis

**DOI:** 10.1371/journal.pone.0197548

**Published:** 2018-05-30

**Authors:** Elizabeth J. Millings, Maria Caterina De Rosa, Sarah Fleet, Kazuhisa Watanabe, Richard Rausch, Dieter Egli, Gen Li, Charles A. Leduc, Yiying Zhang, Stuart G. Fischer, Rudolph L. Leibel

**Affiliations:** 1 Naomi Berrie Diabetes Center and Division of Molecular Genetics, Department of Pediatrics, Columbia University, New York, New York, United States of America; 2 Department of Biostatistics, Mailman School of Public Health, Columbia University, New York, New York, United States of America; Indiana University, UNITED STATES

## Abstract

We have previously reported that *Ildr2* knockdown via adenovirally-delivered shRNA causes hepatic steatosis in mice. In the present study we investigated hepatic biochemical and anatomic phenotypes of Cre-mediated *Ildr2* knock-out mice. Liver-specific *Ildr2* knock-out mice were generated in C57BL/6J mice segregating for a floxed (exon 1) allele of *Ildr2*, using congenital and acute (10-13-week-old male mice) Cre expression. In addition, *Ildr2* shRNA was administered to *Ildr2* knock-out mice to test the effects of *Ildr2* shRNA, *per se*, in the absence of *Ildr2* expression. RNA sequencing was performed on livers of these knockdown and knockout mice. Congenital and acute liver-specific and hepatocyte-specific knockout mice did not develop hepatic steatosis. However, administration of *Ildr2* shRNA to *Ildr2* knock-out mice did cause hepatic steatosis, indicating that the *Ildr2* shRNA had apparent “off-target” effects on gene(s) other than *Ildr2*. RNA sequencing and BLAST sequence alignment revealed *Dgka* as a candidate gene mediating these “off-target” effects. *Ildr2* shRNA is 63% homologous to the *Dgka* gene, and *Dgka* expression decreased only in mice displaying hepatic steatosis. *Dgka* encodes diacylglycerol kinase (DGK) alpha, one of a family of DGKs which convert diacylglycerides to phosphatidic acid for second messenger signaling. *Dgka* knockdown mice would be expected to accumulate diacylglyceride, contributing to the observed hepatic steatosis. We conclude that ILDR2 plays a negligible role in hepatic steatosis. Rather, hepatic steatosis observed previously in *Ildr2* knockdown mice was likely due to shRNA targeting of *Dgka* and/or other “off-target” genes. We propose that the gene candidates identified in this follow-up study may lead to identification of novel regulators of hepatic lipid metabolism.

## Introduction

Non-alcoholic fatty liver disease (NAFLD) is rapidly becoming the leading cause of liver failure and transplantation in the United States and is predicted to affect ~30% of adults in the US [1]. Often considered the major liver manifestation of the metabolic syndrome, NAFLD is closely associated with obesity, diabetes and insulin resistance [1, 2]. While the simple steatosis that defines NAFLD is relatively benign, it can progress to non-alcoholic steatohepatitis (known as NASH) with inflammatory infiltration and fibrosis [3]. The physiologic and metabolic factors that cause NAFLD and trigger its progression to NASH remain poorly understood.

Recently, we described immunoglobulin-like domain containing receptor 2 (ILDR2) as a novel modulator of NAFLD development [4]. Initially identified by positional genetics as a diabetes-susceptibility gene in mice [5], *Ildr2* knockdown via adenovirally-delivered shRNA (ADKD) resulted in gross hepatic steatosis and inflammation within 10 days of infection [4]. Transcript analyses indicated initial increase in expression of genes mediating lipogenesis (3 days post-adenovirus infection), followed by decrease in expression of these transcripts after development of steatosis, and differential expression of genes involved in the unfolded protein response (ER stress) pathways [4].

In this previous study, we used an adenoviral delivery system to target hepatic short hairpin RNA (shRNA) in order to produce an acute liver-specific knockdown of *Ildr2* [4]. In the absence (at the time) of any congenital *Ildr2* KO mouse models, the Adv-shRNA system allowed us to investigate the effects of acute knockdown of *Ildr2* transcripts in the liver. However this system has effects beyond the knockdown of the target gene that confound interpretation: Adv infection is known to trigger hepatic inflammation [6–8] which plays a role in the progression of NAFLD and development of NASH [9, 10]; Adv can also target other tissues, and even though the majority is taken up by the liver [11–13], there are potential consequences for gene expression in those tissues; and, finally, shRNA itself can have “off-target” effects and reduce expression of genes not intentionally targeted [14, 15].

Here we describe liver-specific *Ildr2* gene deletion models achieved using the Cre-loxP system. We discuss the development of liver-specific *Ildr2* knockout (KO) mice and further characterize them to understand the putative role of *Ildr2* in hepatic steatosis. The differing phenotypes observed in *Ildr2* Adv-shRNA KD vs. KO models highlight some of the pitfalls of using adenoviruses and shRNA for genetic manipulations; these are discussed below.

## Results

### Congenital, hepatocyte-specific *Ildr2* KO mice do not develop hepatic steatosis

**Fig 1:**
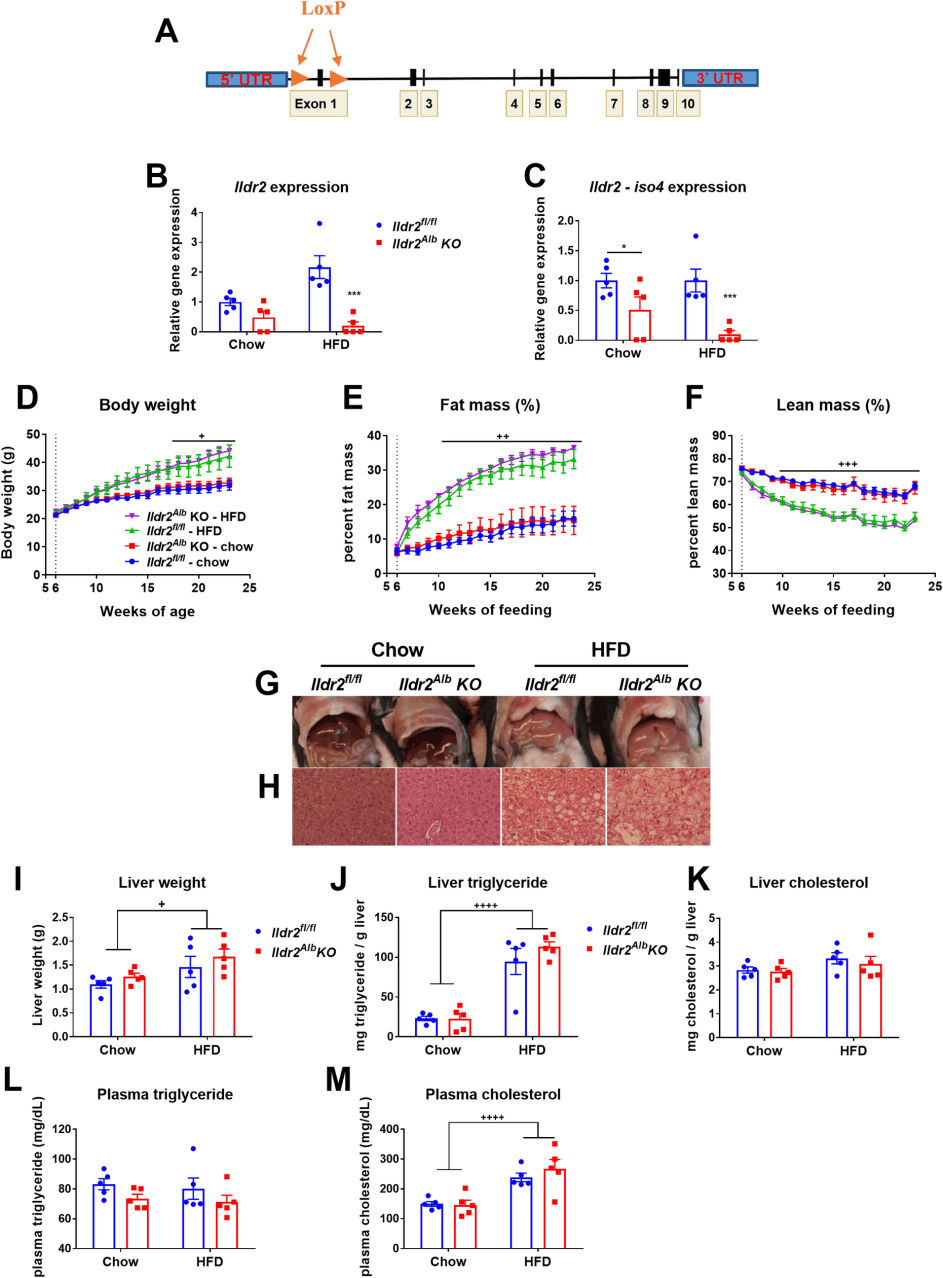
Albumin-cre, *Ildr2* KO mice do not develop hepatic steatosis. (A) Schematic of the floxed *Ildr2* allele (not to scale). (B,C) Expression of *Ildr2*, isoforms 1 and 4 in livers of 23-week old *Ildr2*^*Alb*^ KO mice and littermate *Ildr2*^*fl/fl*^ controls, fed chow or HFD for 17 weeks. Expression was measured by qPCR and normalized to *36b4*, *actb* and *Gapdh* expression. (D) Body weight curves of HFD and chow-fed, *Ildr2*^*Alb*^ KO mice. (E,F) Percent fat mass and lean mass of HFD and chow-fed, *Ildr2*^*Alb*^ KO mice measured weekly by NMR. (G) Photographs of livers excised from HFD and chow-fed, *Ildr2*^*Alb*^ KO mice at 23 weeks of age. (H) Hematoxylin and eosin staining of representative liver sections at 50X magnification. (I) Liver weight at 23 weeks of age. (J,K) Liver triglyceride and total cholesterol content (measured in duplicate). (L,M) Plasma triglyceride and total cholesterol concentration at 23 weeks of age after a 4hr. fast. n = 4–5 mice per group. Data are represented as mean ± standard error (SEM) * p<0.05, ** p<0.01, *** p<0.001 for *Ildr2*^*Alb*^ KO vs. *Ildr2*^*fl/fl*^ control. **+** p<0.05, ++ p<0.01, +++ p<0.001 for chow vs. HFD.

We introduced loxP sites flanking exon 1 of the *Ildr2* gene (exon 1 is included in all seven known *Ildr2* transcript isoforms [5]) to create an *Ildr2* floxed mouse (*Ildr2*^*fl/fl*^) (**Fig 1A**). To explore the function of ILDR2 in the liver, we crossed *Ildr2*^*fl/fl*^ mice with mice expressing Cre recombinase driven by the albumin promoter, obtaining hepatocyte-specific, congenital *Ildr2* knockout mice (see **Table 1** for nomenclature). *Ildr2* liver mRNA expression was reduced >99% in hepatocyte-specific *Ildr2* KO mice (*Ildr2*^*Alb*^ KO) compared to *Ildr2*^*fl/fl*^ littermate controls (**Fig 1B and 1C**). Although a subset of these mice retained *Ildr2* expression–indicating that the albumin-cre was not completely penetrant–these mice displayed no phenotypic differences vs. complete *Ildr2*^*Alb*^ KO mice.

**Table 1:** Mouse models—nomenclature and abbreviations.

Mouse model	Abbreviation in text	Cell type(s) targeted	Developmental Timing	Control used	Phenotype without *Ildr2* shRNA	Phenotype with *Ildr2* shRNA
Adenoviral *Ildr2* shRNA	ADKD	All liver cells	Adult; acute	Adenoviral *lacZ* shRNA		Hepatic steatosis (extreme) and inflammation
*Ildr2*^*fl/fl*^; albumin-cre	*Ildr2*^*Alb*^ KO	Hepatocytes	E14.5 (upon albumin expression)	*Ildr2*^*fl/fl*^	No difference from control	Hepatic steatosis (mild) and inflammation
*Ildr2*^*fl/fl*^; adeno-associated virus-thyroxine-binding globulin (TBG)-cre	*Ildr2*^*AAV*^ KO	Hepatocytes	Adult; acute	*Ildr2*^*fl/fl*^; AAV-TBG-GFP	No difference from control	
*Ildr2*^*fl/fl*^; adenoviral-cre	*Ildr2*^*Adv*^ KO	All liver cells	Adult; acute	*Ildr2*^*fl/fl*^; Adv-GFP	No difference from control	

When fed, ad libitum, low-fat (9% kcal as fat) chow diet, male, *Ildr2*^*Alb*^ KO mice did not differ in body weight or body composition from *Ildr2*^*fl/fl*^ littermates (**Fig 1D–1F**). When fed *ad libitum* a high-fat diet (HFD, 60% kcal as fat) from 6–23 weeks of age, they increased body weight and fat mass in tandem with their *Ildr2*^*fl/fl*^ HFD-fed littermates (**Fig 1D–1F**).

23-week-old, chow-fed *Ildr2*^*Alb*^ KO mice did not exhibit hepatic steatosis by inspection, histology, or quantitative chemical analysis (**Fig 1G–1K**). They also had normal plasma triglyceride and total cholesterol concentrations (**Fig 1L and 1M**). 23-week-old, HFD-fed mice showed hepatic lipid accumulation and elevated plasma lipids, but there was no significant difference between *Ildr2*^*Alb*^ KO mice and littermate controls fed the same HFD (**Fig 1G–1M**).

### Acute, hepatocyte-specific Ildr2 KO mice do not develop hepatic steatosis

The absence of steatosis in *Ildr2*^*Alb*^ KO mice led us to postulate that the congenital nature of the KO may have triggered gene compensation for the lack of ILDR2 during development. The mouse *albumin* gene is turned on at ~E10.5, about halfway through embryonic development [16], and Cre expression has been detected in fetal mouse hepatocytes from albumin-cre mice as early as E14.5, as immature cells begin to differentiate into hepatocytes [17]. Critical genes deleted at this stage in development may be compensated by functionally similar genes [18–20]. Compensation for the loss of *Ildr2* in *Ildr2*^*Alb*^ KO mice could theoretically explain the absence of increased steatosis in the *Ildr2*^*Alb*^ KO mice.

**Fig 2:**
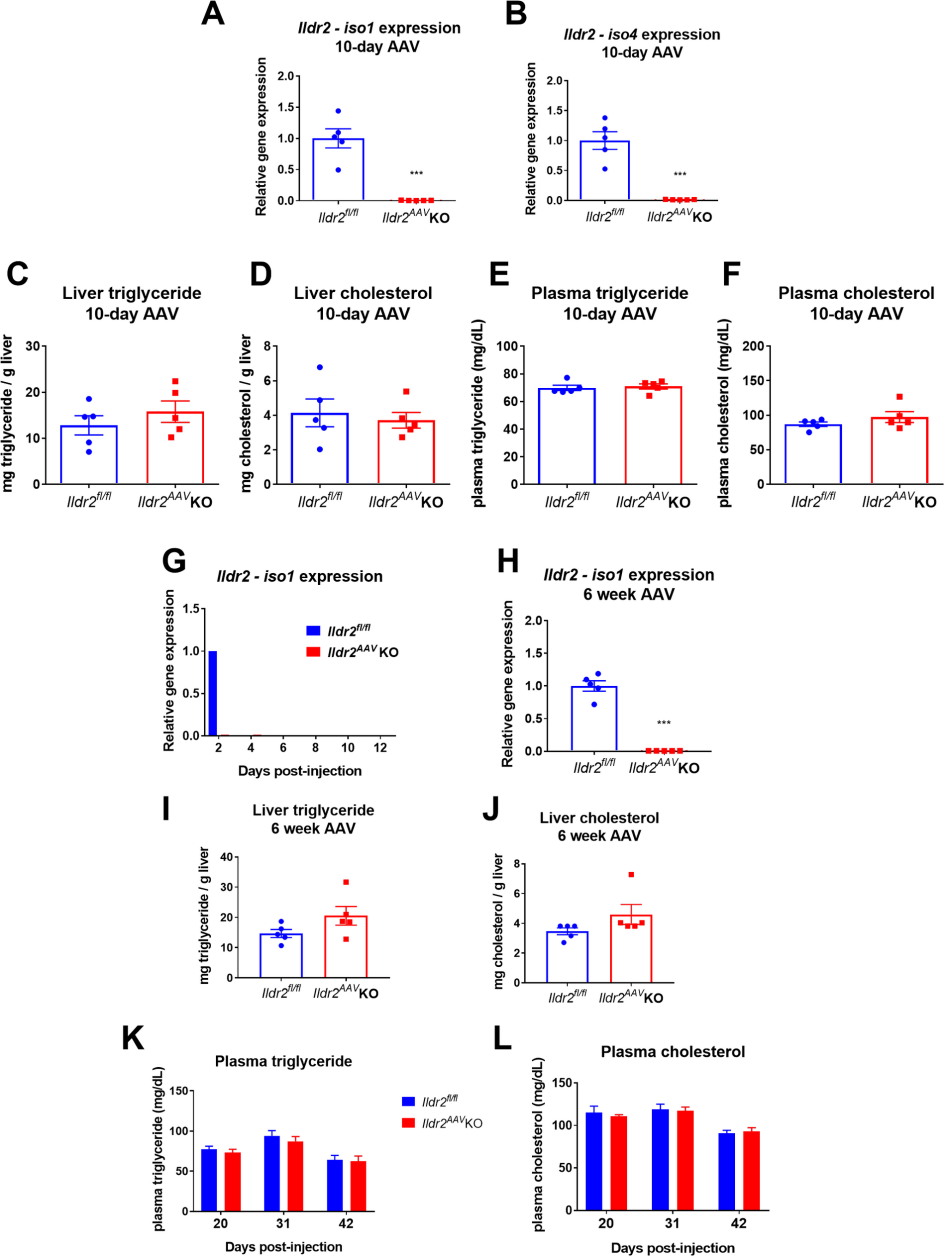
AAV *Ildr2* KO mice do not develop hepatic steatosis. (A,B) qPCR expression of *Ildr2*, isoforms 1 and 4 in livers of 13-week old mice, 10 days after i.v. injection with AAV-TBG-Cre (*Ildr2*^*AAV*^ KO) or AAV-TBG-GFP (*Ildr2*^*fl/fl*^ controls). (C,D) Liver triglyceride and total cholesterol content at 10 days. (E,F) Plasma triglyceride and total cholesterol concentration at 10 days. (G) qPCR expression of *Ildr2* (isoform 1 unless otherwise noted) in livers of 13-week old mice 2–12 days after i.v. injection with AAV-TBG-Cre (*Ildr2*^*AAV*^ KO). AAV-TBG-GFP was only administered for the 2-day timepoint (*Ildr2*^*fl/fl*^ controls). (H) qPCR expression of *Ildr2* in livers of 18-week old mice, 6 weeks after AAV injection (I,J) Liver triglyceride and total cholesterol measurements. (K,L) Plasma triglyceride and total cholesterol concentration at 20, 31 and 42 days after AAV injection. Blood was collected after a 4hr fast. n = 4–5 mice per group. * p<0.05, ** p<0.01, *** p<0.001 for *Ildr2*^*AAV*^ KO vs. *Ildr2*^*fl/fl*^ control.

To address this possibility, we designed a mouse model in which *Ildr2* could be acutely ablated in the adult animals, similar to the original Adv-shRNA KD mice (ADKD) mice. We utilized an adeno-associated virus (AAV) construct incorporating thyroxine-binding globulin (TBG) promoter-driven Cre or GFP (control). This AAV8-TBG-Cre (developed by the Penn Vector Core) enables acute Cre expression specifically in hepatocytes [21, 22], knocking out *Ildr2* (*Ildr2*^*AAV*^ KO). We injected AAV8-TBG-Cre intravenously into 13-week-old, chow-fed *Ildr2*^*fl/fl*^ mice and examined livers 10 days post-injection, in keeping with the timeline of development of steatosis in ADKD mice [4]. Despite complete KO of *Ildr2* (**Fig 2A and 2B**), livers of *Ildr2*^*AAV*^ KO mice were normal, showing neither steatosis nor any lipid metabolic abnormalities when compared to mice injected with the AAV8-TBG-GFP control construct (**Fig 2C–2F**).

To determine the timing of AAV delivery and gene interruption, we measured hepatic mRNA expression of *Ildr2* isoforms 1 through 5 (only isoform 1 is shown) in mice at 2, 4, 6, 8, 10 and 12 days post injection and found that *Ildr2* transcription was eliminated as early as 2 days post-injection (**Fig 2G**). We also followed mice for 6 weeks after AAV infection, measuring plasma lipids at 20 days post-injection, and then at 11-day intervals until sacrifice. *Ildr2*^*AAV*^ KO mice maintained normal plasma lipid levels and did not exhibit any hepatic lipid accumulation or metabolic abnormalities at 6 weeks post-injection with AAV (**Fig 2H–2L**).

### Acute, Adv-mediated, liver-specific *Ildr2* KO mice do not develop hepatic steatosis

Next, we considered the possibility that loss of *Ildr2* in non-parenchymal liver cells may have contributed significantly to the steatosis observed in our original ADKD mice [4]. Both the AAV-TBG-Cre and the albumin-cre were designed to induce recombination and gene knockout specifically in hepatocytes which comprise ~80% of liver tissue. However, the shRNA adenovirus used to produce ADKD mice would have targeted additional liver cell types, such as liver macrophages (Kupffer cells), stellate cells, and epithelial cells. While hepatic steatosis is defined by lipid accumulation in hepatocytes, effects in non-parenchymal liver cells can accelerate the progression of steatosis to more advanced liver disease [23–26]. As resident liver macrophages, Kupffer cells initiate the immune response to metabolic injury, secreting pro-inflammatory chemokines and cytokines such as IL-1β and TNF, stimulating pro-apoptotic signaling pathways in hepatocytes, and recruiting circulating immune cells to the liver [26–28]. Stellate cells play a key role in the induction of fibrosis in liver disease, and can transdifferentiate into myofibroblasts leading to increased production of collagen and extra-cellular matrix (ECM) [25, 28, 29].

**Fig 3:**
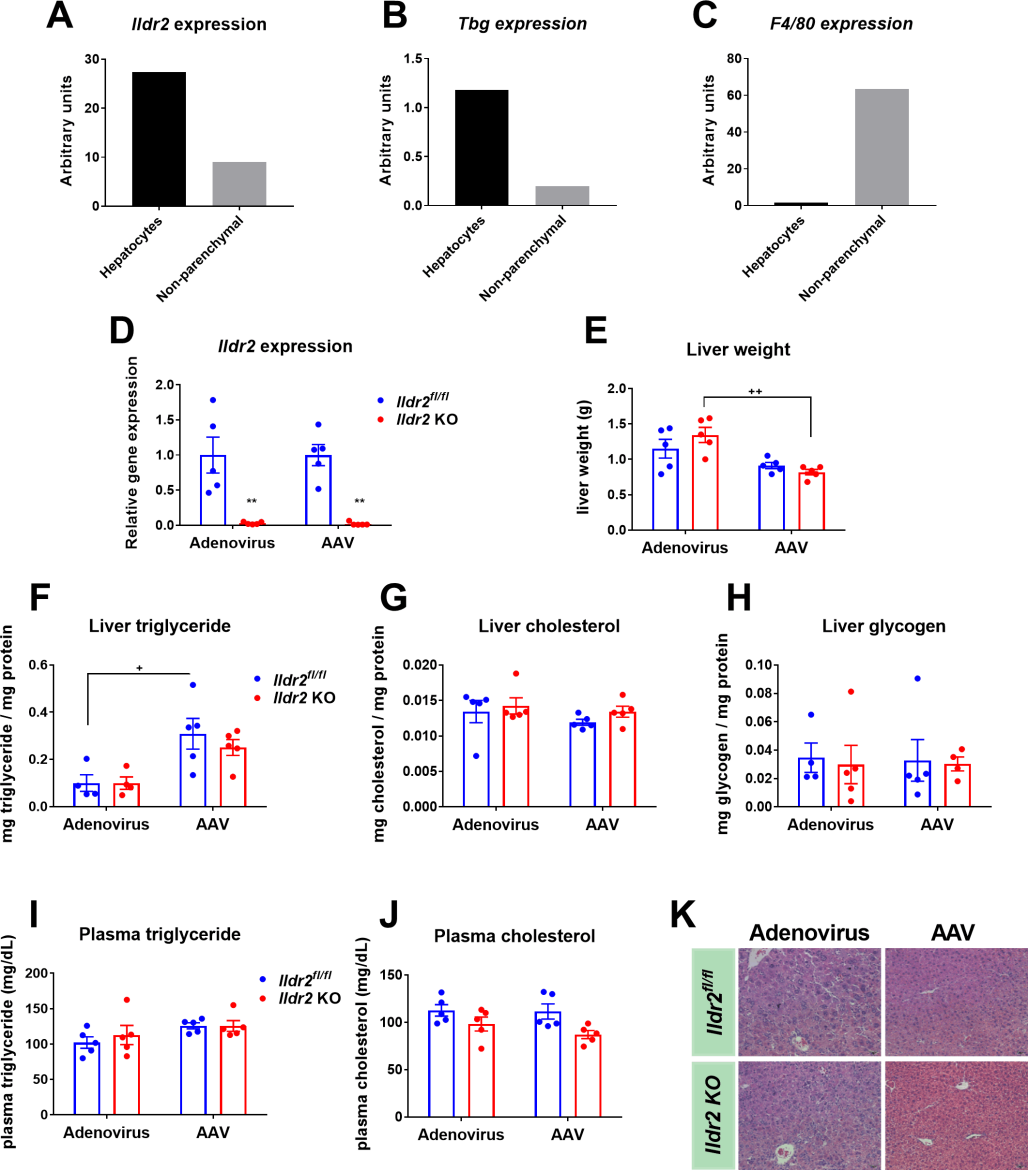
Adenoviral *Ildr2* KO mice do not develop hepatic steatosis. (A-C) qPCR expression of *Ildr2*, *Tbg* and *F4/80* in hepatocyte or non-parenchymal cell fractions isolated from 12-week old wild-type (B6) mice. (D) qPCR expression of *Ildr2* in livers of 11-week old mice, 10 days after i.v. injection with adenoviral-Cre (*Ildr2*^*Adv*^ KO), adenoviral-GFP (*Ildr2*^*fl/fl*^ controls), AAV-TBG-Cre (*Ildr2*^*AAV*^ KO) or AAV-TBG-GFP (*Ildr2*^*fl/fl*^ controls). (E) Liver weights at sac. (F-H) Liver triglyceride, total cholesterol and glycogen content. (I,J) Plasma triglyceride and total cholesterol concentration at sac following a 12hr fast. (K) Hematoxylin and eosin staining of representative liver sections at 20X magnification. n = 4–5 mice per group. * p<0.05, ** p<0.01, *** p<0.001 for *Ildr2* KO vs. *Ildr2*^*fl/fl*^ control. **+** p<0.05, ++ p<0.01, +++ p<0.001 for adenovirus vs. AAV.

To determine if *Ildr2* is expressed in non-parenchymal liver cells, or in hepatocytes only, primary hepatocytes and non-parenchymal cells were isolated from 12-week-old mice using liver collagenase digestion [4]. Hepatocyte and non-hepatocyte cell fractions were separated by centrifugation [30]. Gene expression analysis of liver cell markers was used to confirm the cellular identity of each fraction. *Tbg*, a hepatocyte-specific marker, and *F4/80*, a macrophage-specific marker, were highly expressed in the hepatocyte and non-hepatocyte fractions, respectively (**Fig 3B and 3C**). *Ildr2* was expressed in both cell fractions, although at about one-third the level in the non-hepatocyte cell fraction as in the hepatocyte cell fraction (**Fig 3A**). However, since these cell fractions were sorted by centrifugation there could be cross-contamination as indicated by low-level *Tbg* expression in the non-hepatocyte fraction, and *F4/80* expression in hepatocyte fraction (**Fig 3B and 3C**). Microarray expression data from Xu, *et al*. also confirm that *Ildr2* is expressed in various populations of macrophages derived from adipose tissue [31]. Taken together, these results suggest that *Ildr2* ablation in non-parenchymal liver cells could contribute to the steatotic phenotypes of the ADKD mice, and thus explain the lack of hepatic steatosis in the acute and congenital transgenic hepatocyte-specific KO mice.

To test this possibility, we created another acute *Ildr2* KO model by using an adenoviral-Cre construct rather than the AAV-TBG-Cre used previously. While the AAV-TBG-Cre construct is designed to impact only hepatocytes, adenoviral-Cre targets both parenchymal and non-parenchymal liver cells [32, 33]. 11-week-old, male, *Ildr2*^*fl/fl*^ mice were injected intravenously with adenovirus-Cre or adenovirus-GFP as a control. Age-matched *Ildr2*^*fl/fl*^ mice were infected with AAV-TBG-Cre or AAV-TBG-GFP at the same time for parallel comparison. *Ildr2*^*Adv*^ KO mice were euthanized 10 days post-injection. No liver steatosis or dyslipidemia were seen in *Ildr2*^*Adv*^ or *Ildr2*^*AAV*^ KO mice despite complete *Ildr2* ablation in liver (**Fig 3D and 3F–3J**). *Ildr2*^*Adv*^ KO livers were heavier compared to *Ildr2*^*AAV*^ KO mice (**Fig 3E**), and also showed histological evidence of inflammation (**Fig 3K)**. However, as these phenotypes were also present in the *Ildr2*^*Adv*^ GFP control mice, we attributed them to the effects of adenovirus treatment as has been documented previously [6–8].

### Administration of adenoviral *Ildr2* shRNA causes TG accumulation in *Ildr2*^*Alb*^ KO mice

We have produced three distinct models of hepatic *Ildr2* KO: a congenital, hepatocyte-specific KO (*Ildr2*^*Alb*^ KO); an acute, hepatocyte-specific, KO (*Ildr2*^*AAV*^ KO); and an acute, liver-specific KO (*Ildr2*^*Adv*^ KO). None of these models showed the severe steatohepatitis observed in the adenoviral *Ildr2* shRNA (ADKD) model [4]. Thus, we were driven to consider that the steatosis had been caused by a consequence of the shRNA antisense construct–primarily unrelated to the decrease in *Ildr2* expression.

The original ADKD model was produced by treating mice with an adenovirally-delivered shRNA. Thus, the adenovirus treatment and/or the shRNA itself may have triggered liver steatosis. Since we showed that adenoviral treatment alone does not cause hepatic steatosis, so we turned our attention to the *Ildr2* shRNA. This shRNA was specifically designed to target exon 2 which is present in all isoforms of *Ildr2* mRNA; however, the construct may have had “off target” effects on other genes as discussed below [14, 15, 34].

**Fig 4:**
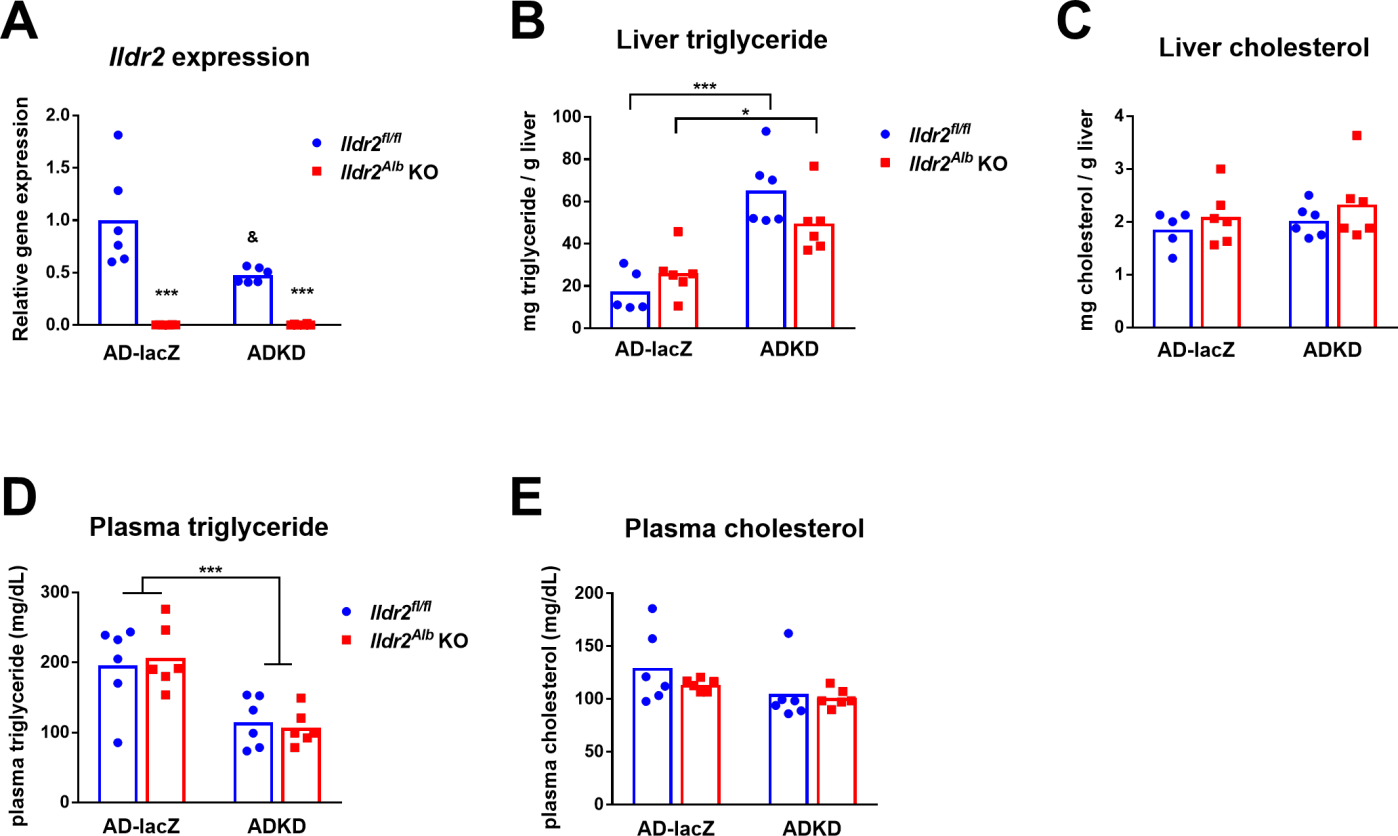
Effects of adenoviral *Ildr2* shRNA in *Ildr2* KO mice. (A) qPCR expression of *Ildr2* in livers of 10-week old mice *Ildr2*^*Alb*^ KO mice and littermate *Ildr2*^*fl/fl*^ controls, 10 days after i.v. injection with ADKD or AD-lacZ. (B,C) Liver triglyceride and total cholesterol content. (D-E) Plasma triglyceride and total cholesterol concentration at sac following a 24hr fast. n = 5–6 mice per group. * p<0.05, ** p<0.01, *** p<0.001 for *Ildr2*^*fl/fl*^ vs. *Ildr2*^*Alb*^ KO; & p<0.05 for ADKD vs. AD-lacZ.

To determine if other targets of the shRNA contributed to the KD liver phenotype, we infected *Ildr2*^*Alb*^ KO mice with the original KD adenoviral shRNA [4]. Since these mice do not express *Ildr2* in the hepatocytes, any steatosis observed would be the result of shRNA targeting of other genes affecting lipid metabolism. 10-week-old, male, *Ildr2*^*Alb*^ KO or *Ildr2*^*flfl*^ control mice were injected intravenously with the original adenovirus expressing *Ildr2* shRNA (ADKD), or with control adenovirus expressing lacZ shRNA (AD-lacZ) [4]. Mice were euthanized at 10 days post-adenovirus infection following a 24-hour fast. Gene expression analysis by qPCR confirmed that *Ildr2* was completely ablated in *Ildr2*^*Alb*^ KO mice, regardless of Adv treatment (**Fig 4A**). In *Ildr2*^*flfl*^ mice, *Ildr2* shRNA (ADKD) reduced *Ildr2* mRNA by about 50% vs. AD-lacZ treated *Ildr2*^*flfl*^ mice (**Fig 4A**). We did not observe gross liver steatosis, but chemical quantification of hepatic lipid content revealed that ADKD-treated mice had significantly increased hepatic TG compared to AD-lacZ treated mice, across both genotypes (3-fold in *Ildr2*^*flfl*^, 1.5-fold in *Ildr2*^*Alb*^ KO) (**Fig 4B and 4C**). Conversely, plasma TG was significantly decreased in *Ildr2* shRNA treated *Ildr2*^*Alb*^ KO and *Ildr2*^*flfl*^ mice vs. AD-lacZ treated mice for both genotypes, although plasma cholesterol was unchanged (**Fig 4D and 4E**).

These results confirm that the *Ildr2* shRNA is sufficient to cause hepatic steatosis despite the preexisting absence of *Ildr2*. *Ildr2* expression was reduced by 50% in *Ildr2* shRNA *Ildr2*^*flfl*^ mice, indicating that acute partial loss of *Ildr2* expression might contribute to the development of steatosis. However, the degree of steatosis and hypotriglyceridemia did not differ between *Ildr2* shRNA *Ildr2*^*flfl*^ and *Ildr2* shRNA *Ildr2*^*Alb*^ KO mice, suggesting that *Ildr2* expression is either irrelevant to the phenotype or has an equivalent effect at levels below a specific threshold, i.e. below 50%. In either case, the major trigger for hepatic steatosis is the *Ildr2* shRNA, not *Ildr2* ablation *per se*.

These results suggest that the *Ildr2* shRNA also targets other gene(s) involved in hepatic lipid metabolism, and that KD of these gene(s) is primarily responsible for the gross steatosis in the original *Ildr2* shRNA ADKD mice [4] as well as the less striking, but still significantly increased TG accumulation observed here.

### RNAseq analysis of *Ildr2* shRNA ADKD vs. *Ildr2*^*Adv*^ KO livers reveal candidate genes for shRNA off-target effects on hepatic steatosis

The *Ildr2* shRNA we used was designed to target exon 2 of the *Ildr2* mRNA, which is present in all known *Ildr2* isoforms. Analysis of the 19 base pair (bp) shRNA sequence, GTTCAAATCCTACTGCCAG, using NCBI BLAST (Basic Sequence Alignment Search Tool) identified no perfectly matched sequences (other than *Ildr2*) leaving open the possibility that partially matched sequence(s) could be targeted and cause knockdown of gene(s) essential for hepatic lipid homeostasis [15, 34].

To identify additional gene(s) that might have been knocked down by the *Ildr2* shRNA, and thus have contributed to development of steatosis in ADKD mice, we performed RNA sequencing analysis on liver samples from ADKD and AD-lacZ mice (from our previously published ADKD study [4]) and *Ildr2*^*Adv*^ KO mice (**Fig 3**). ADKD and AD-lacZ samples were harvested 3 days post Adv infection to increase the likelihood of detection of primary effects of knocking down the gene rather than secondary gene changes resulting from hepatic steatosis *per se*.

**Fig 5:**
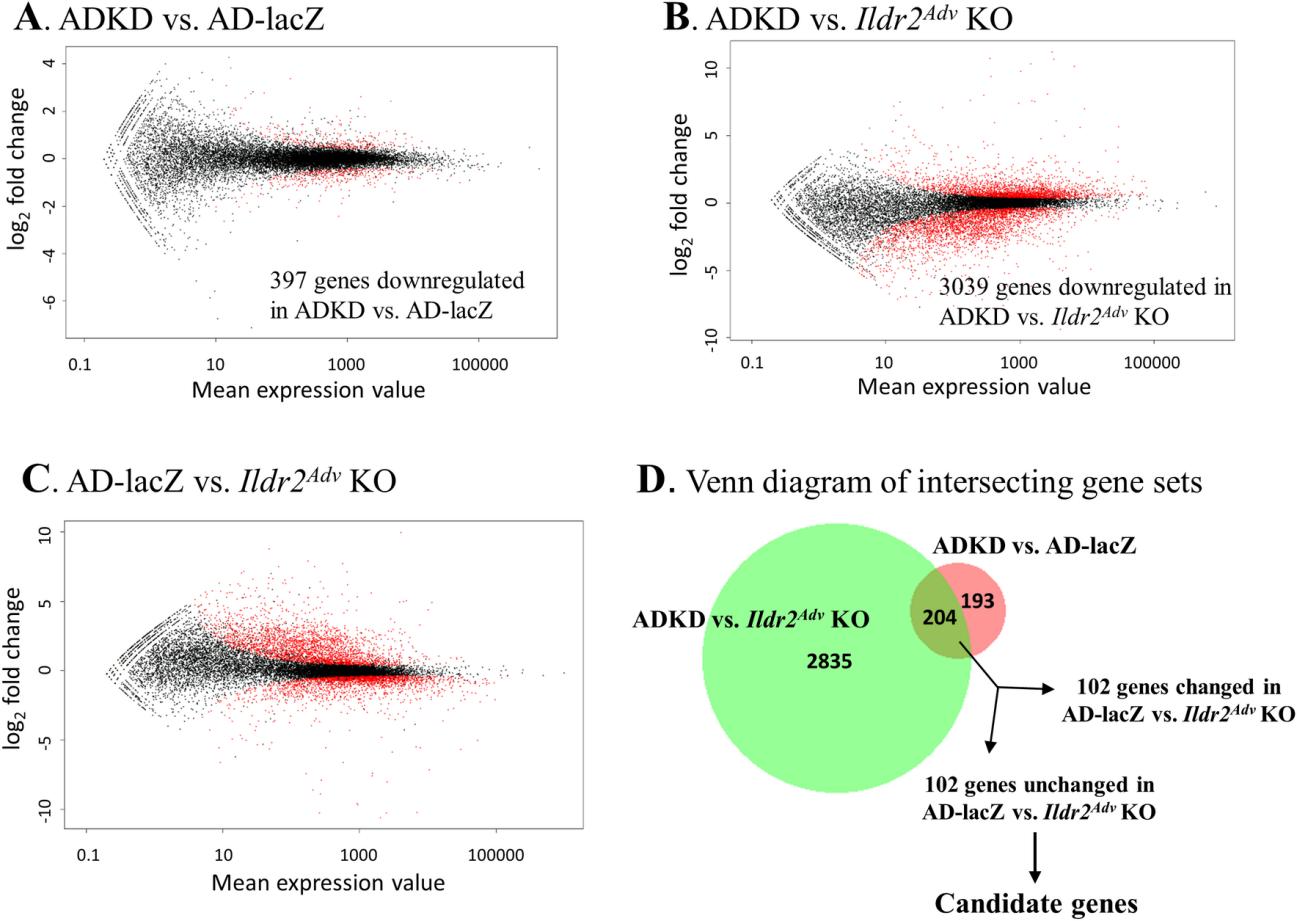
RNAseq analysis of AdV-KD vs. *Ildr2*^*Adv*^ KO livers identifies candidate genes for shRNA off-target effects. “Minus over average” (MA) plots showing log_2_ fold change vs. normalized mean for each comparison. Red dots represent significantly upregulated or downregulated genes in (A) ADKD vs. AD-lacZ mice, (B) ADKD and *Ildr2*^*Adv*^ KO mice, (C) AD-lacZ vs. *Ildr2*^*Adv*^ KO mice. (D) Venn diagram illustrating how the 102 candidate genes were identified. The intersection of genes downregulated in ADKD mice vs. *both* AD-lacZ and *Ildr2*^*Adv*^ KO was 204. 102 of these genes were not significantly changed in AD-lacZ vs. *Ildr2*^*Adv*^ KO. These became the target gene candidates (see **S1 Table** for complete list). This Venn diagram was created using BioVenn software [37] (www.biovenn.nl).

RNAseq count expression data were analyzed with *DEseq*, a differential expression analysis program based on the negative binomial distribution [35]. Pairwise comparisons were made between *Ildr2* shRNA (ADKD) and AD-lacZ; *Ildr2* shRNA ADKD and *Ildr2*^*Adv*^ KO; and AD-lacZ and *Ildr2*^*Adv*^ KO. The Benjamini-Hochberg adjustment [36] was used to correct for the multiple testing problem. **Fig 5A–5C** are “minus over average” (MA) scatter plots of differential gene expression profiles for each of the 3 comparisons. MA plots display the entire gene set, comparing fold change between samples (y-axis) to mean expression value (x-axis) with significantly differentially expressed genes highlighted in red (**Fig 5A–5C**). We screened for candidate genes that were: 1) significantly decreased in ADKD vs. AD-lacZ; 2) significantly decreased in ADKD vs. *Ildr2*^*Adv*^ KO; and, 3) not significantly changed between AD-lacZ and *Ildr2*^*Adv*^ KO livers, indicating a specific effect of the KD *Ildr2* shRNA shRNA (**Fig 5D**). Using these parameters, we identified 102 candidate genes (**S1 Table**).

These candidates were further refined by searching for genes that have been implicated in NAFLD genome-wide association studies (*Ppp1ca*) [38], genes associated with other liver disease, (*Dguok*, *Ass1*) [39, 40], and obesity-related genes (*Slc39a1*) [41]. Since the shRNA was targeted to *Ildr2*’s exon 2 which encodes for an IgG domain, we identified genes that are part of the IgG-like family (*Neo1*, *Ptp4a1*, *Scn8a*, *Unc13b*); additionally, we found a gene located near *Ildr2* on chromosome 1 (*Pogk*) [5].

Initial BLAST searches of the shRNA sequence yielded no complete match apart from *Ildr2*. However, searching for truncated portions of the 19-bp sequence yielded a partial match in *Dgka*. *Dgka* is one of the 102 candidate genes identified by RNAseq analysis (**S1 Table**), and is 63% homologous to the *Ildr2* shRNA sequence. The first 12 bp of the 19 bp shRNA sequence, GTTCAAATCCTA, exactly a sequence in exon 4 of the *Dgka* mRNA. *Dgka* expression is downregulated by 50–60% in ADKD livers compared to AD-lacZ and *Ildr2*^*Adv*^ KO samples, suggesting that it could be targeted by the *Ildr2* shRNA. *Dgka* encodes diacylglycerol kinase alpha (DGKα), which functions to convert diacylglycerides (DAGs) to phosphatidic acid [42]. DAG species are increased in mouse embryonic fibroblasts from *Dgka*-null mice [43] similar to increases in DAG species observed in steatotic human liver [44]. These data suggest that loss of *Dgka* expression could result in the steatosis observed in our ADKD mice.

## Discussion

In this study we describe several mouse models developed in an effort to replicate the hepatic steatosis phenotype of adenoviral *Ildr2* shRNA KD mice. Using the Cre-loxP system, we created congenital and acute, hepatocyte-only and liver-specific *Ildr2* KO mice. However, none of these KO models recapitulated the phenotype of hepatic steatosis observed in the adenoviral *Ildr2* shRNA KD mice [4].

RNAi-mediated knockdowns have been effectively used in many experimental settings, and are particularly useful in *in vitro* studies, and in instances in which a genetic knockout would be prohibitively expensive or difficult to make, or where the knockout is embryonically lethal. KD and KO models are generally quite similar, e.g., *Pparα* siRNA KD mice phenocopy the null transgenics [45] and *Cx43* KO and KD mouse astrocytes have very similar transcriptional profiles [46].

However, discrepancies between RNAi-mediated KD and KO mouse models are not uncommon. As observed in this study and by others, siRNAs and shRNAs can have off-target effects due to sequence similarity to unintended gene targets [14, 15, 34]. RNAi-mediated knockdowns can also exhibit a more severe phenotype than the KO or null mutant due to disruption of the gene at a more mature developmental stage, when functional compensation is difficult [18, 47]. This situation has been documented, for example, for the genes *thymosin β4* and *Sprn*/*Prnp* in mice, and *ABP1* in *Arabdopsis thaliana* [48–50].

Our studies in which *Ildr2* shRNA KD adenovirus was administered to *Ildr2*^*Alb*^ KO mice revealed that lipid accumulation occurred with the adenovirus treatment, regardless of *Ildr2* genotype of the recipient mouse (**Fig 4**). These experiments indicate that the hepatic lipid phenotype is due primarily to treatment with the adenovirus shRNA, rather than to loss of *Ildr2* expression *per se*, which suggests that this shRNA targeted genes in addition to *Ildr2*. We identified *Dgka*, among other gene candidates, as a potential target of adenoviral *Ildr2* shRNA in ADKD mice. Given its homology to the shRNA sequence, its reduced expression in ADKD mice, and its functional role in lipid metabolism [43, 44, 51], we propose that shRNA targeting of *Dgka* could account for the difference in lipid accumulation between ADKD and KO mice.

Adenovirus is an efficient vector for introduction of gene products into cells both *in vitro* and *in vivo*. The adenovirus used in this and our previous study [4] was human adenovirus serotype 5, one of the most commonly used adenoviruses which displays specific liver tropism, and thus is very useful for directing gene products to the liver. However, use of adenoviruses in these contexts can be problematic for several reasons. One overriding concern is that, since they are infectious agents, they can stimulate inflammatory responses in the infected cells [6–8]. This response can mask or confound the effect(s) of whatever biological molecules are being delivered to the cells. Another issue with adenovirus is that its tissue tropism, while specific, is not exclusive, and it can affect tissues other than the target tissue [11]. Additionally, the various methods of measuring adenoviral titer make it difficult to control the amount of active virus that is administered in an experiment, which can lead to significant variation among experiments. The sensitivity of viral activity to temperature changes, i.e. freeze-thaw cycles, also contributes to experimental variability [52, 53].

The experiments described here highlight some of the difficulties in working with adenoviruses. In addition to possible aberrant RNAi gene targeting, the striking phenotype of the original ADKD mice may also have been due to adenovirus-induced inflammation and /or targeting of extra-hepatic tissues. While we confirmed that *Ildr2* expression was maintained in other tissues of ADKD mice [4], we cannot rule out that the Adv may have infected other organs. Another concern is that the amount of active *Ildr2* shRNA adenovirus used to infect *Ildr2*^*Alb*^ KO mice may have decreased from its activity level at the time of ADKD infection. Although the same titer was used in both experiments (3x10^11^ optical particle units (OPU)/mouse), this titer only measures adenovirus concentration, not viral activity. A reduction in adenoviral activity could also explain the difference in lipid accumulation and severity of steatosis between ADKD mice and *Ildr2*^*Alb*^ KO mice infected with Adv-*Ildr2* shRNA. Use of appropriate controls enabled us to deconvolute the effects of *Ildr2* expression on hepatic steatosis in our various mouse models; however, the confounding effects of using adenovirus as a primary delivery system remain a significant issue.

## Conclusions

We have clearly shown that loss of *Ildr2*—whether specifically in hepatocytes or in all liver cells—is not sufficient to cause hepatic steatosis. These studies indicate that, contrary to the inferences reached based on acute shRNA-mediated KD [4], ILDR2 plays a minimal role in hepatic lipid metabolism. We propose that interruption of other gene(s) significantly contributed to the steatotic phenotype of the original ADKD. RNA-seq identified 102 genes that are significantly reduced in ADKD mice vs. *Ildr2*^*Adv*^ KOs or AD-lacZ controls (**S1 Table**). Among these 102 genes, the most likely candidate is *Dgka*, which contains a sequence that could be targeted by the *Ildr2* shRNA. It is worth noting that hepatic lipid accumulation due to Adv-*Ildr2* shRNA treatment was only observed in mice with at least a 50% reduction in *Ildr2* expression. Thus, it is possible that KD of candidate gene(s) interacts with *Ildr2* hypomorphism to induce steatosis.

*Ildr2* was initially identified as modifier of diabetes susceptibility [5], and ongoing work in our lab has confirmed its role in beta cell function, glucose homeostasis, and metabolic partitioning of energy stores. Additionally, ILDR2, along with ILDR1 and ILDR3, are members of the angulin family which maintain membrane integrity at tricellular epithelial tight junctions [54, 55]. Our development of conditional KO mice to clarify the role of ILDR2 in the liver, can now facilitate the study of ILDR2 in various tissues and conditions, enabling a more complete understanding of this novel gene in mammalian biology.

## Methods

### Ethics statement

All animal experiments were approved by Columbia Institutional Animal Care and Use Committee (Protocol# AAAH0707 and AAAR0416).

### Animal care

Mice were housed at room temperature in a 12-hr light/12hr-dark vivarium, with ad libitum access to 5058 Purina PicoLab Mouse Diet 20 (9% kcal from fat) and water. High-fat diet (HFD) fed mice received chow with 60% kcal from fat (Research Diets #D12492i). Where noted, blood was collected by submandibular bleeding. Fat and lean mass were measured with an EchoMRI Analyzer (Bruker Optics), calibrated using mouse carcasses [56]. Mice were euthanized by cervical dislocation for liver tissue collection or prior to primary hepatocyte isolation.

### Mouse strains

To generate *Ildr2* floxed mice (*Ildr2*^*fl/fl*^), a targeting construct containing *Ildr2* exon 1 flanked by loxP sites and a neomycin resistance cassette (transcribed in opposite direction of gene, 1.6 kb from exon 1) was electroporated into V6.5 (129 x B6 F1) ESCs. Electroporated ESCs were selected with G418, picked, and 384 colonies were clonally expanded. Positive integration of the targeting construct was identified by PCR followed by confirmation of correct integration by Southern blot. A positive clone was injected into E3.5 BDF1 blastocytes and transferred into the uteri of pseudopregnant CD1 mice (Crl:CD1; Charles River). Floxed alleles in the pups were confirmed by PCR. Chimeric mice were crossed with C57BL/6J (Jackson Labs) mice and offspring were confirmed carriers by PCR and Southern blot analysis. Mice that carried the floxed allele were backcrossed to C57BL/6J mice (obtained from Jackson Labs) for 10 generations then intercrossed to create B6.129S-*Ildr2*^*fl/fl*^ mice. These mice were bred with albumin-Cre mice (B6.Cg-Tg(Alb-cre)21Mgn/J, Jackson Labs stock #003574) until all offspring segregated for two floxed alleles and one or no copies of Cre.

### Adenovirus production and administration

Adenovirus expressing *Ildr2* shRNA was designed, produced and amplified as previously described [4]. Adenovirus expressing *lacZ* shRNA was designed and produced as previously described [4], but amplification and purification procedures were performed by Welgen, Inc (Worcester, MA). Mice were administered 3x10^11^ OPU/mouse via tail vein injection. AAV-TBG-Cre, AAV-TBG-eGFP, adenoviral-Cre, and adenoviral-GFP were obtained from the University of Pennsylvania Vector Core (Philadelphia, PA). Mice were administered 1.3x10^11^ genome copies/mouse via tail vein injection.

### Lipid measurements in tissue and plasma

Capillary blood from submandibular bleeds was collected in heparinized tubes and centrifuged at 200 x *g* for 20 minutes at 4°C to separate plasma. Lipid extraction from liver was adapted from the Folch method [57]. Approximately 100 mg tissue were homogenized in 3 mL phosphate-buffered saline (PBS). 12 mL 2:1 chloroform: methanol (CHCl_3_: MeOH) were added and mixture was vortexed twice for 15 seconds each. After centrifuging at 3000 rpm for 10 minutes, the organic lower layer was transferred to a 20-mL glass scintillation vial. An additional 10 mL 2:1 CHCl_3_: MeOH were added to upper layer and vortexing and centrifugation were repeated. Organic lower layer was added to first extraction in scintillation vial. Solvent was dried down under nitrogen (N_2_) gas followed by lipid resuspension in 1 mL 15% Triton X-100 in CHCl_3_. Solvent was dried down again under N_2_ gas and remaining lipid was resuspended in 1mL H_2_O. Triglyceride and total cholesterol in plasma and liver extracts were measured with the Infinity Triglycerides (Thermo Scientific) and Cholesterol E (Wako Diagnostics) kits, respectively.

### Glycogen measurement

For glycogen extraction 100 mg tissue were homogenized in 1 mL H_2_O on ice, boiled for 10 minutes, then centrifuged at 13,000 x *g* for 10 minutes to pellet insoluble material. Supernatant was transferred to a new tube and used for glycogen measurement. Glycogen was measured using a glycogen assay kit from Sigma-Aldrich (#MAK016)

### Primary hepatocyte and non-parenchymal cell isolation

Primary hepatocytes isolation was performed as previously described [4] with the exception of euthanasia prior to laparotomy and perfusion. The supernatant from primary hepatocyte centrifugation was collected and spun down at 500 x *g*, for 10 minutes at 4°C according to a protocol for isolating Kupffer cells by Xu, et al. [30]. The pelleted cells from this centrifugation were considered the non-parenchymal cell fraction.

### Hematoxylin and eosin histology

Liver sections were fixed in aqueous zinc-buffered formalin (Anatech, Ltd.), sectioned and visualized by hematoxylin (Fisher) and eosin (Crystalgen) staining. Images were obtained using an Olympus IX73 inverted microscope (Olympus America).

### RNA extraction, reverse transcription and quantitative PCR

Tissue and cell samples were homogenized in TRIzol® Reagent (Invitrogen) and extracted using the TRIzol® reagent protocol or the PureLink™ RNA Mini kit (Invitrogen). Reverse transcription was performed using the Transcriptor First Strand cDNA Synthesis kit (Roche). qPCR was performed using a Roche LightCycler® 480 instrument. qPCR primers are listed in **Table 2**. Tissue-specific standard curves for each gene (primer pair) were used to convert threshold crossing point (Cp) values to relative concentrations, which were then normalized to *36b4*, *Actb*, and/or *Gapdh* expression. In instances in which standard curves were not used, Cp values are shown, with lower Cp values indicating greater mRNA expression.

**Table 2:** List of qPCR primers.

Mouse gene name	Forward primer (5’ to 3’)	Reverse primer (5’ to 3’)
*36b4*	ACCTCCTTCTTCCAGGCTTTGG	CGAAGGAGAAGGGGGAGATGTT
*Actb*	CGGGCTGTATTCCCCTCCAT	GGGCCTCGTCACCCACATAG
*Gapdh*	CTGGAGAAACCTGCCAAGTATGATG	GAGACAACCTGGTCCTCAGTGTAGC
*Ildr2 –isoform 1*	GATTATGCCAGAGTGGGTGTTTGTC	CCCTGCTTCATACAAGGCCTGAG
*Ildr2 –isoform 4*	AACAGGGCTCGACGGTTAC	AACACCCACTCCAACACCAG
*Tbg*	GCAGAAAGGATGGGTTGAATTG	AAGTCAGCACTTTCAGCAAAGG
*F480*	CTTTGGCTATGGGCTTCCAGTC	GCAAGGAGGACAGAGTTTATCGTG

### RNAseq

RNA was extracted from liver samples as detailed above and sample integrity was assessed with an Agilent 2100 Bioanalyzer with all samples having RIN numbers greater than 8.0. mRNA was isolated using a poly-A pulldown [58] and reverse transcription to generate cDNA. The cDNA was sequenced using single-ended sequencing on a HiSeq2000 according to manufacturer’s recommendations (Illumina; San Diego, CA). The pass filter (PF) reads were mapped to mouse reference genome mm9 using TopHat (version 2.0.4). TopHat infers novel exon-exon junctions *ab initio*, and combines them with junctions from known mRNA sequences (refgenes) as the reference annotation [59]. For each read, we allowed up to 3 mismatches and 10 multiple hits during the mapping. The software package DESeq [35] in R (Version 3.4.1) was used to conduct the differential gene expression analysis. Multiple comparisons were adjusted by the Benjamini-Hochberg approach, and differentially expressed genes were determined at false discovery rate of 0.01.

### Statistical analysis

Statistics were performed using GraphPad Prism 7 software. Data are represented as mean ± standard error (SEM). Statistical comparisons were made using the Student’s two-tailed t-test for 2 groups or two-way analysis of variance (ANOVA) for 4 groups.

## Supporting information

S1 Table RNAseq candidate gene list. (PDF)
