# Supplementary material for: ILDR2 has a negligible role in hepatic steatosis
Source: PLoS One. 2018 May 30;13(5):e0197548. doi: 10.1371/journal.pone.0197548 (PMC5976177; doi:10.1371/journal.pone.0197548)
Supplement: S1 Table — (PDF) [file pone.0197548.s001.pdf]

S1 Table: RNAseq candidate gene list

	Average counts			p_{adj} values		
**Gene name**	**AD-lacZ**	**ADKD**	***Ildr2**^{Adv}* **KO**	**ADKD vs. AD-lacZ**	**ADKD vs. *Ildr2**^{Adv}* **KO**	**AD-lacZ vs. *Ildr2**^{Adv}* **KO**
Rps19bp1	276	66	267	6.98E-28	6.49E-18	0.199
Slc39a1	1314	422	1524	2.18E-26	1.42E-31	1.000
Slc27a4	1167	473	1504	3.05E-23	1.04E-08	0.666
Aldh4a1	5277	2521	6117	3.63E-19	9.96E-05	1.000
Ndst1	3091	1464	3079	8.28E-19	1.37E-10	0.127
Ppp1ca	3562	1720	4107	4.12E-18	1.20E-15	0.956
Rab1b	3043	1523	3700	4.45E-16	3.10E-16	0.882
2900097C17Rik	1326	662	1417	2.73E-14	5.36E-10	0.478
Ptp4a1	529	246	558	4.76E-13	1.85E-06	0.463
6330578E17Rik	2685	1434	2716	5.05E-13	2.98E-07	0.174
Tbc1d13	852	426	1090	1.00E-12	5.29E-15	0.571
Ubfd1	972	494	1155	1.28E-12	3.30E-12	1.000
Gm98	1354	708	1630	1.54E-12	1.30E-12	0.948
Tmem123	1090	563	1404	2.37E-12	5.18E-15	0.520
Nt5dc2	336	151	374	1.68E-11	2.06E-09	0.819
Neo1	671	345	676	7.21E-11	1.09E-05	0.224
Sumf2	291	135	290	9.58E-10	8.90E-06	0.283
Acpl2	266	124	338	4.16E-09	9.22E-11	0.711
Pcgf2	181	79	210	2.44E-08	2.32E-04	1.000
Cpsf2	783	448	841	4.44E-08	9.82E-06	0.539
Wfdc2	201	59	190	5.30E-08	6.97E-09	0.260
Ccdc50	1569	946	2019	7.78E-08	1.72E-10	0.518
Tspan4	626	359	772	1.70E-07	1.80E-04	0.876
Reck	81	27	87	4.09E-07	6.94E-06	0.776
Pef1	1057	642	1345	4.99E-07	3.00E-09	0.599
Scpep1	1018	618	1254	5.33E-07	2.74E-08	0.814
Diablo	560	326	548	7.33E-07	2.44E-03	0.148
Icmt	1088	675	1172	1.65E-06	1.36E-04	0.535
Celf1	3216	2074	3415	1.96E-06	4.12E-04	0.405
Dgka	**160**	**75**	**187**	**2.38E-06**	**1.57E-06**	**1.000**
Ssu72	986	613	1306	2.66E-06	1.26E-09	0.360

Stat6	1590	1019	1825	4.42E-06	1.33E-05	0.946
Chtf8	1247	792	1459	4.68E-06	5.19E-06	1.000
Dpp8	1734	1118	1816	5.03E-06	1.21E-03	0.352
Ipo5	1941	1256	2423	5.06E-06	2.05E-04	0.748
Snx4	802	500	980	6.65E-06	6.53E-07	0.884
1810011O10Rik	785	491	927	7.77E-06	4.37E-06	1.000
Trappc9	395	232	451	8.09E-06	2.62E-05	0.930
Erp29	949	602	1228	8.70E-06	2.19E-08	0.489
Eif3h	2005	1332	2224	2.32E-05	3.12E-04	0.712
Pja2	1062	693	1459	3.17E-05	1.63E-09	0.191
Arl4d	531	312	756	4.58E-05	8.90E-05	0.370
Cdt1	233	121	366	5.32E-05	6.90E-05	0.207
Rab34	205	97	260	6.48E-05	3.52E-09	0.749
Pogk	155	82	225	7.69E-05	6.56E-09	0.216
Gtf3c1	1771	1062	1764	9.27E-05	1.34E-03	0.140
Pofut1	410	255	540	1.02E-04	2.24E-07	0.454
Atr	601	389	716	1.29E-04	8.64E-05	1.000
Tbc1d20	807	534	894	1.38E-04	1.04E-03	0.733
Rpa2	295	155	341	1.44E-04	6.00E-04	1.000
Nol3	24	4	36	1.49E-04	1.04E-07	0.533
Psmd14	1404	955	1472	1.54E-04	9.94E-03	0.359
Tax1bp3	72	30	82	1.58E-04	2.18E-04	0.969
Tab2	1916	1221	2563	1.77E-04	2.59E-10	0.272
Cenpm	148	62	167	1.77E-04	6.02E-07	0.915
Adss	1756	1163	2309	1.97E-04	5.07E-04	0.602
Zdhhc2	178	74	216	1.97E-04	2.57E-04	1.000
Ern1	836	554	886	2.51E-04	5.07E-03	0.454
Sox12	129	68	142	2.55E-04	3.50E-03	0.850
Slc4a4	3541	2086	3876	2.74E-04	5.33E-07	0.621
Setd8	1595	1039	1698	2.80E-04	1.04E-03	0.440
S100a16	385	245	425	2.90E-04	1.66E-03	0.753
Rassf5	334	210	429	3.19E-04	4.41E-06	0.623
Tmem189	331	208	364	3.42E-04	1.95E-03	0.751
Plod3	447	290	618	3.49E-04	6.00E-08	0.220
Scn8a	38	12	46	4.04E-04	1.18E-04	1.000
Pcnxl3	1147	795	1261	5.32E-04	4.14E-03	0.677
Akap11	1114	771	1316	5.49E-04	2.44E-04	1.000

2310022B05Rik	445	291	599	5.60E-04	5.46E-07	0.336
Vps39	584	391	754	5.91E-04	4.03E-06	0.543
Nacc1	1123	783	1361	7.35E-04	9.58E-05	0.911
Zswim7	182	107	196	7.37E-04	5.19E-03	0.680
Entpd5	11076	7174	13493	7.81E-04	9.33E-08	0.877
Ass1	4516	2710	8270	8.68E-04	2.85E-03	0.133
Pip4k2c	656	449	870	9.94E-04	1.80E-06	0.382
Dguok	231	144	250	1.15E-03	6.84E-03	0.693
Hfe	1107	783	1321	1.41E-03	4.08E-04	1.000
Atg16l2	212	133	238	2.03E-03	3.74E-03	0.892
P4hb	21192	15697	28758	2.26E-03	1.49E-05	0.181
Ctsz	4039	2972	4668	2.40E-03	2.43E-03	0.977
P4ha2	131	59	153	2.61E-03	2.92E-03	1.000
Slc34a2	47	13	38	2.86E-03	6.03E-03	0.350
Nle1	85	45	123	3.35E-03	5.77E-06	0.358
Grina	3684	2736	4162	3.76E-03	8.64E-03	0.835
Gnl3l	445	311	630	5.38E-03	8.05E-07	0.155
Unc13b	258	132	309	5.41E-03	3.97E-06	0.998
Tesk1	476	333	563	5.89E-03	1.98E-03	1.000
Mcm3	371	248	546	6.06E-03	4.12E-03	0.346
Maged1	2143	1603	2458	6.93E-03	8.08E-03	0.938
Frmd8	522	359	738	6.97E-03	1.74E-07	0.139
Eaf1	1713	1276	2136	7.07E-03	7.08E-04	0.725
Inppl1	1205	890	1596	7.12E-03	1.70E-05	0.351
4732418C07Rik	478	338	563	7.12E-03	3.10E-03	1.000
Slk	1319	980	1676	8.11E-03	1.49E-04	0.606
Comtd1	99	57	151	8.13E-03	1.72E-06	0.163
Dock8	823	553	1011	8.68E-03	1.93E-05	0.848
Pdia4	4016	2883	4695	8.70E-03	5.53E-04	1.000
Echdc3	948	700	1165	9.08E-03	7.53E-04	0.828
Galns	352	246	490	9.20E-03	6.04E-06	0.228
1110008P14Rik	390	275	474	9.37E-03	1.52E-03	0.928
Edem1	3313	2515	4237	9.87E-03	1.16E-04	0.533
AI597479	322	223	410	9.87E-03	3.30E-04	0.684
